# Bedrock sculpting under an active alpine glacier revealed from cosmic-ray muon radiography

**DOI:** 10.1038/s41598-019-43527-6

**Published:** 2019-05-06

**Authors:** R. Nishiyama, A. Ariga, T. Ariga, A. Lechmann, D. Mair, C. Pistillo, P. Scampoli, P. G. Valla, M. Vladymyrov, A. Ereditato, F. Schlunegger

**Affiliations:** 1 Albert Einstein Center for Fundamental Physics, Laboratory for High-Energy Physics, University of Bern, Bern, Switzerland; 2 Earthquake Research Institute, The University of Tokyo, Tokyo, Japan; 3 Faculty of Arts and Science, Kyushu University, Fukuoka, Japan; 4 Institute for Geological Sciences, University of Bern, Bern, Switzerland; 5 Dipartimento di Fisica “E.Pancini”, Università di Napoli Federico II, Naples, Italy; 6 Institut des Sciences de la Terre – CNRS, Université Grenoble Alpes, Grenoble, France

**Keywords:** Geomorphology, Particle physics

## Abstract

Mountain glaciers form landscapes with U-shaped valleys, roche moutonées and overdeepenings through bedrock erosion. However, little evidence for active glacial carving has been provided particularly for areas above the Equilibrium Line Altitude (ELA) where glaciers originate. This is mainly due to our lack of information about the shape of the bedrock underneath active glaciers in highly elevated areas. In the past years, the bedrock morphology underneath active glaciers has been studied by geophysical methods in order to infer the subglacial mechanisms of bedrock erosion. However, these comprise surveys on the glaciers’ surface, from where it has been difficult to investigate the lateral boundary between the ice and the bedrock with sufficient resolution. Here we perform a muon-radiographic inspection of the Eiger glacier (Switzerland, European Alps) with the aid of cosmic-ray muon attenuation. We find a reach (600 × 300 m) within the accumulation area where strong lateral glacial erosion has cut nearly vertically into the underlying bedrock. This suggests that the Eiger glacier has profoundly sculpted its bedrock in its accumulation area. This also reveals that the cosmic-ray muon radiography is an ideal technology to reconstruct the shape of the bedrock underneath an active glacier.

## Introduction

Glaciers play an important role in limiting the height of mountain ranges and in shaping alpine-type landscapes, which are commonly characterized by U-shaped valleys, cirques and steep-edged ridges along their thalwegs^1–4^. Glaciers deepen and widen pre-existing valleys through processes referred to as abrasion and quarrying/plucking^1,2^. Theory and observations predict that glacial erosion is proportional to the sliding velocity raised to some power^5,6^. In addition, the sliding velocity has been considered to depend on both the basal shear stresses and the fluid pressure ratio, where higher values result in either an increase (shear stress) or a decrease (fluid pressure) in the erosion rates^7,8^. Modern erosion rates under glaciers have been studied from sediment yields and provenance tracing of material in subglacial streams^6,9^. The erosional efficiency of glaciers is known to vary greatly depending on location, climate, strength of the bedrock, energy gradient and ice thickness^1–7,10^. In this context, a major question arises about the potential of extrapolating short-term observations to the timescales over which glacial landscape with typical U-shaped cross-sectional valleys and stepped longitudinal profiles form, which typically develop over multiple glacial cycles (i.e. tens to hundreds of thousand years)^5,11^. Although various numerical models with a particular focus on subglacial erosion have been performed to reproduce glacial landscapes^5,7,12^, these rely strongly both on our knowledge of the ice rheology and the ice-flow mechanisms, as well as on observational data of the glacial bedrock morphology. This potential lack of knowledge on the details of the erosional mechanisms and the underlying physical controls also concerns the questions of how glacial cirques are formed, and how subglacial processes actively sculpt the underlying bedrock^13–16^ in these high-elevation regions.

A key information for improving our understanding of the landscape response to glacial erosion is offered by the bedrock topography from past-glaciated areas and beneath present-day active glaciers, mainly because the bedrock topography directly reflects the erosional patterns and mechanisms at work underneath a glacier^7,17,18^. This has been the major motivation for (1) exploring the bedrock topography using drilling information to map the landscape response to past glaciations^19^; and (2) employing several geophysical techniques including seismic surveys and gravity measurements, ground penetrating radar surveys, and topographic modelling to reconstruct the bedrock topography of formerly glaciated areas or underneath active glaciers^20–27^. Despite the progress of such technologies, the bedrock morphology along the sides of a glacier, particularly in remote high-elevation alpine areas, has been hardly constrained, because these methods are mainly performed from above the glaciers’ surfaces. In addition, most of the alpine cirques are hardly accessible particularly in their accumulation areas where they originate. Where surveys were possible, the resolution of the data decreases rapidly towards the glaciers’ bases and lateral sides. This is related to (1) the presence of non-consolidated sediment deposits on the glacier margin and (2) the steep dip of the bedrock surface underlying most of the surveyed glaciers. In addition, in the case of temperate glaciers, high fluid pressure ratios lower the resolution of seismic and radar surveys^28,29^, thereby thwarting a high-resolution reconstruction of the bedrock shape underneath modern glaciers.

**Figure 1:**
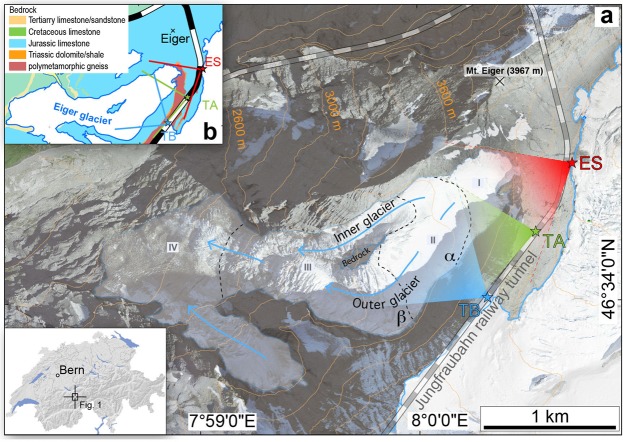
Overview of the study site in the Central Swiss Alps. (**a**) Eiger glacier with distinct morphological domains (I to IV; see main text for discussion) and railway tunnel. Subsurface detector sites (ES: Eismeer station, TA: tunnel site A, TB: tunnel site B) are given with the corresponding view field. (**b**) Simplified geological map^40^ illustrating the main rock types of the study area. The topographic data of both figures has been reproduced with permission by swisstopo (BA18111).

In the present work we apply the cosmic-ray muon radiography technology to map the lateral margin of the Eiger glacier (Fig. 1) situated in Switzerland (Central European Alps at 46°34′05″N latitude and 7°59′56″E longitude). This method is based on the high penetration power of cosmic-ray muons that hit the Earth’s surface continuously^30^, where the attenuation rate of the muon flux mainly depends on the density of the traversed material^31^. The idea dates back to 1955 with the measurements of the thickness of rock above an underground tunnel^32^, followed by the pyramid’s inspection by L. Alvarez in 1970’s^33^, and it has spread to multiple fields such as volcanology^34,35^, geology^36^, and the non-destructive inspection of reactors^37^ in the past decade. In 2015, Nishiyama *et al*.^31^ launched a pilot survey for the application of such a technology in an Alpine environment using emulsion films as muon detectors. These authors^31^ successfully mapped a small portion of the bedrock underneath the uppermost part of the Aletsch glacier (Central European Alps) over an area of c. 50 × 100 m^2^. Based on the success of this pilot work, the present study aims at imaging of a much larger region where a glacier originates and where active erosion and bedrock sculpting are likely to occur.

Muon radiography requires muon detectors to be placed at an elevation lower than the surveyed target because of the downward going path of cosmic rays. We benefited from the railway tunnel of the Jungfraubahn, which runs through the bedrock surrounding and beneath the headwall reaches of the Eiger glacier (Fig. 1). In 2017, we installed muon detectors made of emulsion films at three sites along this tunnel (sites ES, TA and TB, Fig. 1). Emulsion films, which have been applied in various experiments on fundamental physics^38,39^, record the trajectories of incoming muon particles, which can be observed with optical microscopes as sequences of tiny silver grains after chemical development^38^. The microscopic analysis of the films allowed the attenuation pattern of muon intensity to be measured^38^, which in turn gives the information on the density of overburden material^31^. The observed density information is then quantitatively converted to the location of the boundary separating the low-density ice from the high-density bedrock^31^, providing a unique and high-resolution reconstruction of the bedrock topography at the bottom and lateral margins of the Eiger glacier.

## Results

### Morphology of the Eiger glacier

We explored the morphology of the Eiger glacier, which is the target of our survey (Fig. 1). The nearly 2.1 km^2^ large glacier originates on the western flank of Mt. Eiger at approximately 3600 m a.s.l., from where it flows and terminates at an elevation of approximately 2400 m a.s.l. over a length of c. 2 km (Fig. 1). The bedrock geology of the study area comprises a suite of NW-dipping recrystallized micritic limestones that display a penetrative horizontal foliation and that overly polymetamorphic gneisses (inset Fig. 1b)^40^. The bedrock has thus a constant fabric and comprises the same lithology within the entire glacial catchment^40^. The mean value of the bulk density of the bedrock in this region is 2.68 g cm^−3^, determined from 16 bedrock samples^31^, and its standard deviation is 0.02 g cm^−3^. The Eiger glacier consists of four morphologic domains I-IV (Fig. 1): (I) a headwall reach forming a concave amphitheatre-shaped cirque with c. 50° steep flanks (including the headwalls), (II) a relatively flat domain, and (III) a middle segment where a prominent bedrock ridge forces the ice flow to diverge over c. 250 m distance before converging again farther downslope. There, the bedrock ridge, which most likely corresponds to a “roche moutonée” (i.e. a distinctive bump of 10–100-m scale typical of glacially-scoured landscapes with smooth abraded slopes facing upglacier, and abrupt slopes on its downglacier face), is exposed in the middle of the glacier, and the ice surface in segment (iii) is rugged and dissected by several transverse crevasses. Segment (III) also evidences a drastic change in ice-flow direction from South-West to West. The exposed bedrock ridge separates the Eiger glacier in two parts: a slip-off, steep margin situated on the inner northern-side of the glacier (referred hereafter to the “inner glacier”; Fig. 1), and a cut-bank, flatter glacier part on its southern outer margin (referred hereafter to the “outer glacier”; Fig. 1). The down-slope end of segment III also lies within the altitudinal range of the modern ELA, which is situated at c. 3000 m a.s.l.^41^. Finally, segment IV is characterized by a terminal lobe with longitudinal crevasses and a till.

A closer inspection of the glacier morphology discloses further details, particularly along segment II where the Eiger glacier starts to separate into two branches. Downslope of segment II where the ice-flow bifurcation begins, the ice-surface strongly differs between the inner and outer glaciers. The inner glacier has a straight ice flow, with a relatively small ice surface, and the surface elevation rapidly drops from 3400 to 3100 m a.s.l. over a short distance of c. 650 m, yielding in an average surface slope of c. 25°. Contrariwise, the outer glacier follows a large bend where the ice-flow orientation changes by 90° within a few hundred meters. Along this bend, the ice-surface elevation drops of about 300 m over a distance c. 1000 m. Interestingly, this glacier segment is also the location where cuts into its bedrock wall are visible, forming two secondary concave niches *α* and *β* with a shape that is similar to an incipient cirque (Fig. 1). While the upper niche *α* is situated slightly upstream the bend and only weakly developed, the lower niche *β* is located within the bend and characterized by a distinct concavity. The ice surface maintains a constant dip of c. 10° above the 90°-direction bend, and then steepens to c. 22° farther downslope after the flow direction has changed to the West. At this point, transverse crevasses suggest that the ice is under extension, while the absence of any crevasses along the flatter segments implies that the glacier is under compression. Visual inspection shows that the outer glacier has a surface area of c. 300,000 m^2^ and is thus >200% larger than the inner glacier (Fig. 1).

### Muon attenuation pattern

**Figure 2:**
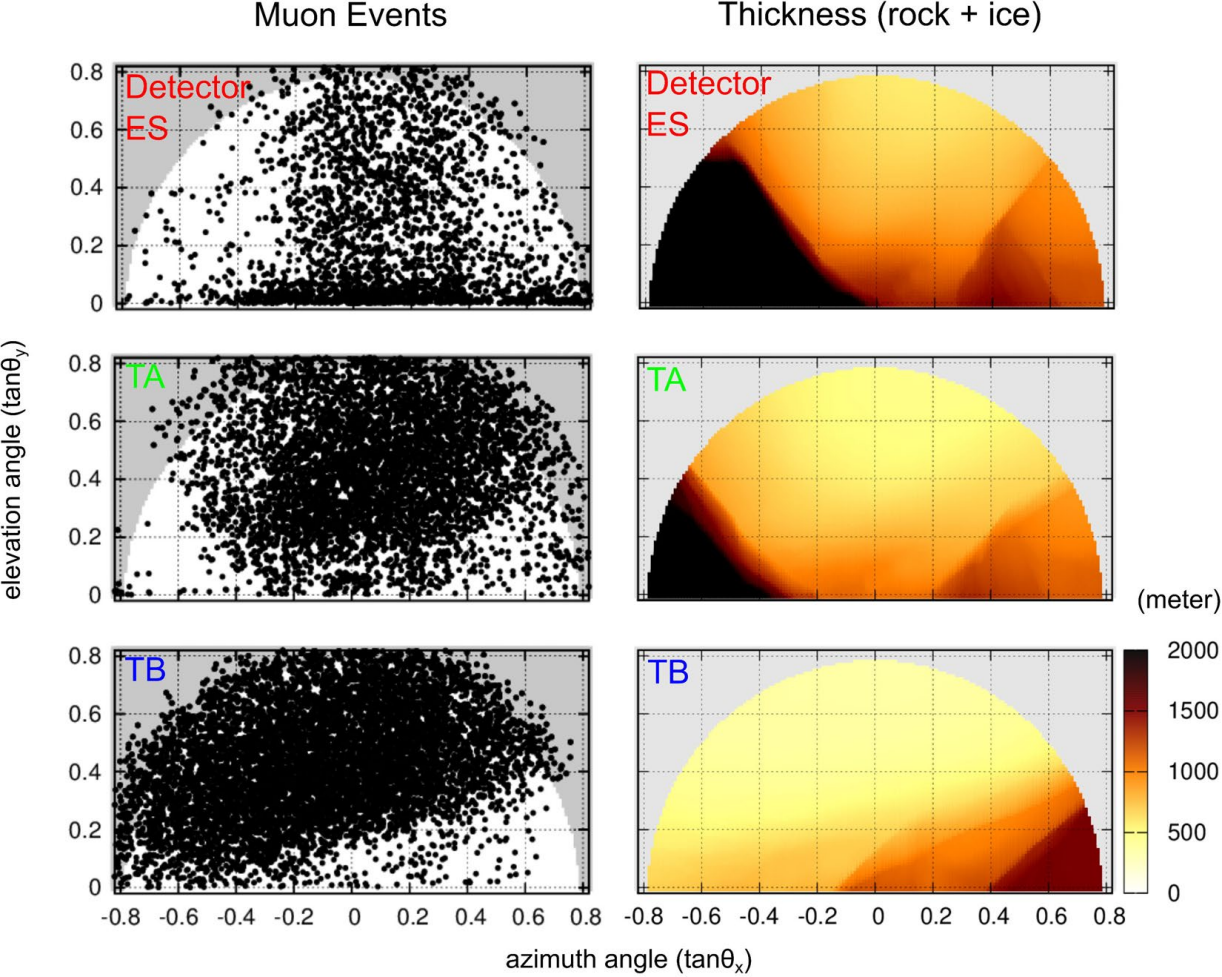
Distribution of recorded muons. Angular distribution of observed tracks at site ES (top), TA (middle) and TB (bottom) with muon trajectories displayed on the left and edifice thickness (ice and/or bedrock) corresponding to muon trajectories displayed on the right. The grey region (left panels) indicates the limit of the effective angular space of the microscope. The muon events in these regions are thus not used for the attenuation quantification.

Figure 2 shows the direction of arriving muons at the three detector sites (ES = Eismeer Station, TA = Tunnel site A and TB = Tunnel site B; Fig. 1). In these diagrams, the direction is represented as azimuth and elevation angles (*θ*_*x*_, *θ*_*y*_). The number of observed muons are 2.3, 5.3 and 7.9 × 10^3^ for ES (exposure time: 106.8 days, effective area: 1512 cm^2^), TA (164.9 days, 1296 cm^2^) and TB (106.8 days, 1080 cm^2^) sites, respectively. The population of the recorded muons is clearly anti-correlated with the thickness of the bedrock and the ice along the straight muon trajectories (Fig. 2). Specifically, the shadows on each plot where the data density is low (sparsely distributed data points) coincide with the regions where the obstacle thickness is greater than 1 km. This indicates that most of the muons from these directions were absorbed in the thick mountain edifice. We binned these registered muons in a rectangular histogram (*tanθ*_*x*_, *tanθ*_*y*_) and converted the data into a normalized flux (cm^−2^ sr^−1^ sec^−1^).

**Figure 3:**
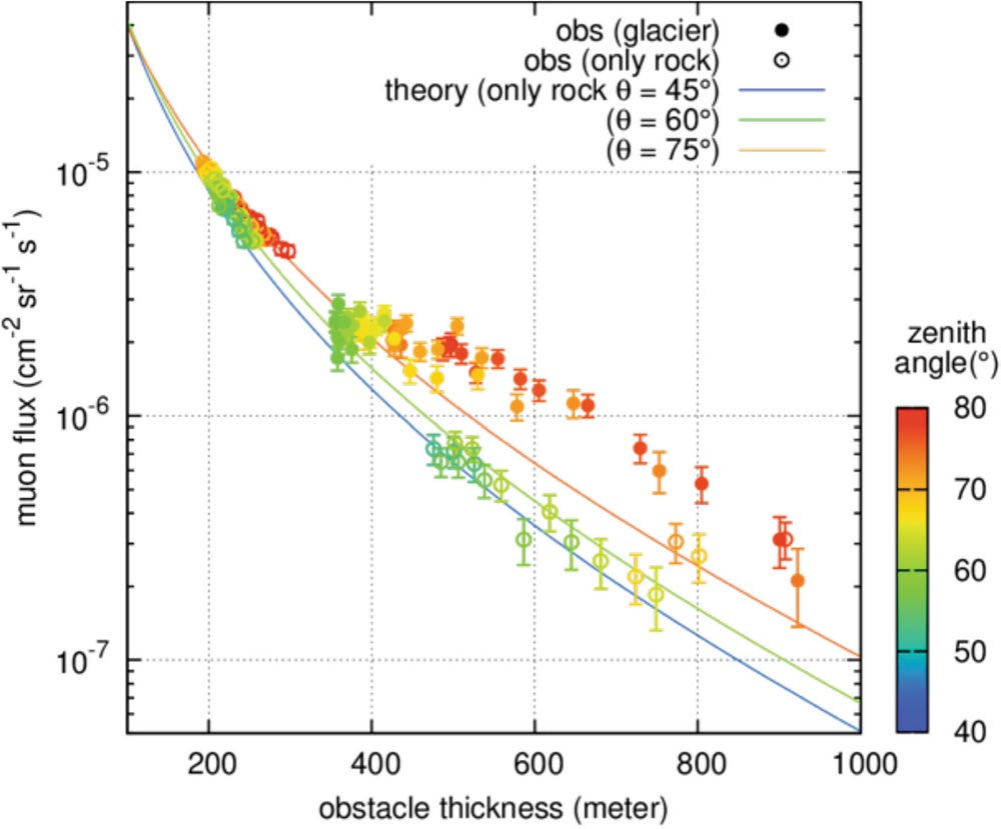
Muon fluxes. Attenuation of muon flux (vertical axis) as a function of the obstacle thickness (horizontal axis). The colours of the data point represent the zenith angle of muons arriving at the detector. The open circles denote muons, which passed only through the bedrock and were observed at the TA site detector. These data are used for calibration purposes (see Method section) by comparing them with the theoretical predictions of the flux attenuation in pure rock (density 2.68 g cm^−3^) reported as solid lines for different zenith angles (45°, 60° and 75°). The solid circles denote muons, which crossed both the ice and the underlying bedrock (displayed data are the ones from the TB site detector).

Figure 3 illustrates how the muon flux data is used to infer the density of the traversed material. First, we focus on the flux data of muons, which passed purely inside the bedrock without crossing the glacier. Such data are available from the TA detector (Fig. 3: open circles, and Fig. 1 for location of detector). As seen in Fig. 3, the flux decreases nearly exponentially with the increase of the bedrock thickness, and its attenuation depends also on the zenith angle of the muons (represented by the colours of the data points). These features of the muon flux attenuation in the bedrock are well reproduced by the simulation for different zenith angles (45°, 60° and 75°) under the assumption of a uniform bulk rock density of 2.68 g cm^−3^ (Fig. 3: solid curves)^31^. This agreement assures that the variety of the bulk density inside the bedrock is small, as is observed with the standard deviation of the density sampling (0.02 g cm^−3^). On the other hand, trajectories where muons cross partially the glacial ice before arriving at the detectors yield flux values that are by a factor of up to 2 higher than these theoretical calculations (Fig. 3: solid circles). This excess of the muon flux is due to the lower bulk density of ice compared to that of bedrock, thus allowing a higher muon transmission. We benefitted from these differences in muon attenuation between ice and rock: the magnitude of the muon flux attenuation recorded at the different tunnel sites can be quantitatively interpreted in terms of the ice-bedrock proportion along the muons’ trajectories^31^, which is then converted into the position of the boundary between the basal ice and its underlying bedrock. The three detectors surrounding the glacier provided information about the bedrock shape from different perspectives. The bedrock positions were first estimated for individual detectors, and the results are combined and re-sampled into a three-dimensional representation of the interface geometry (see Method section).

### Reconstruction of the bedrock shape

**Figure 4:**
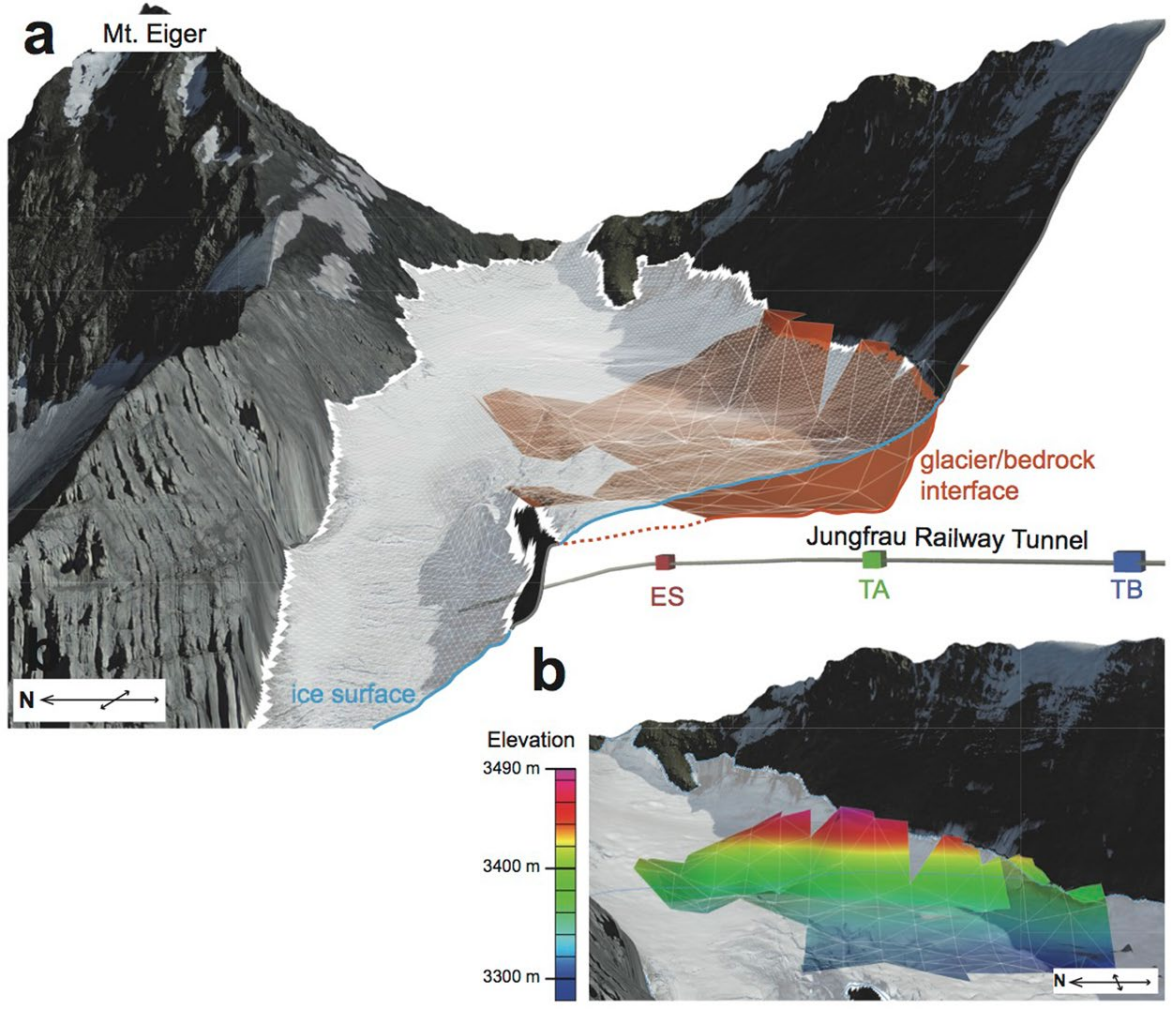
Bedrock topography underneath the Eiger glacier. (**a**) Grid segments that were imaged by the muons at the three detector sites. (**b**) 3D representation of the reconstructed bedrock underneath the Eiger glacier, in which the colours indicate the altitude of the boundary between the glacial ice and the underlying bedrock. The topographic data has been reproduced with permission by swisstopo (BA18111) with a different view.

**Figure 5:**
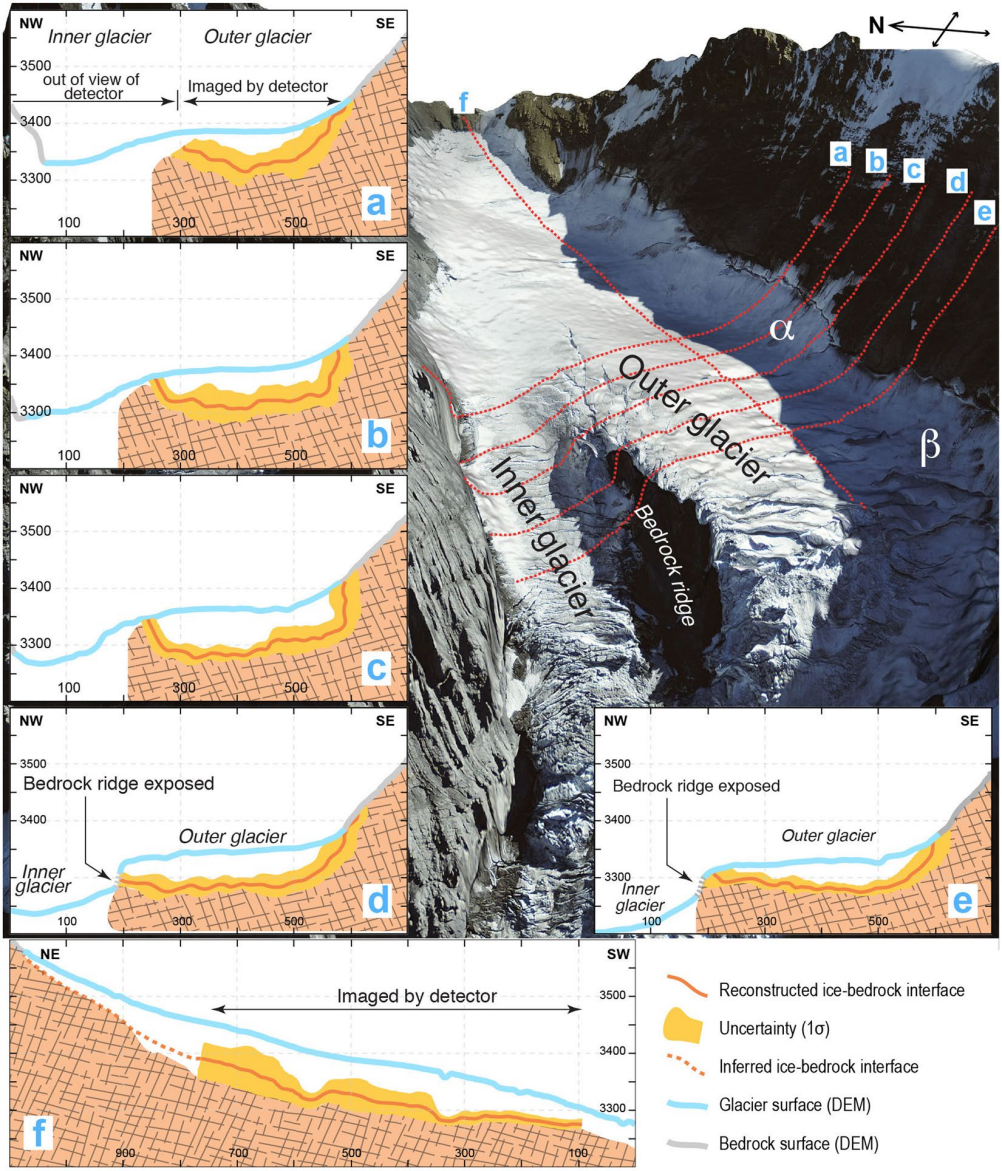
Cross-sections illustrating the bedrock geometry underneath the Eiger glacier. Cross-sectional views of the reconstructed bedrock under the Eiger glacier, along five parallel locations (**a**) to (**e**) perpendicular to the flow direction, and one (**f**) parallel to the flow direction. Orange curves show the best-fitted bedrock positions and yellow bands show associated uncertainties (1σ) due to the statistical fluctuations of muon events. Grey and blue curves represent the hillslope topography and the ice surface. See general view (right) for location of cross-sections. The topographic data has been reproduced with permission by swisstopo (BA18111).

We determined the bedrock shape underneath the Eiger glacier over a 600 m-long (NE-SW) and 300 m-wide reach (NW-SE) (Fig. 4). This corresponds to the segment where the glacier surface is relatively flat and where it dips at c. 10° (segment II on Fig. 1) just below the >30°–50° steep headward reach (segment I on Fig. 1). The elevation resolution of the inferred bedrock surface ranges from 10 m (1σ) in vertical dimension along the relatively flat segment (which is very close to the TB detector), to about 30 m (1σ) in the headward reach where statistics are deteriorated due to the thick mountain edifices. The inferred glacier thickness is typically around 50 m (down-slope segment II on Fig. 1) to 100 m (up-slope segment I on Fig. 1). Along the thalweg axis, the bedrock surface continuously dips in the downslope direction, and neither an overdeepening nor transverse bedrock knobs could be observed (Figs 4 and 5).

The cross-sectional views (Fig. 5) present quantitative information on the bedrock shape beneath the Eiger glacier, particularly on the southern outer side of the bedrock ridge. In the up-slope reach (segment II), represented by cross-section (a) on Fig. 5, the bedrock steeply dips at a constant angle >50° from the lateral valley wall above the glacier down to 50 m beneath the ice surface. There, the transition to the flat glacier base is gradual without any distinct break-in-slope. The situation markedly changes farther down-slope where the boundary between the aerial and subglacial lateral margin is characterized by a sharp break-in-slope (sections (b) and (c) on Fig. 5). There, the dip angle of the bedrock surface changes from c. 50° above the glacier to sub-vertical at the ice contact. In addition, cross-section (c) suggests that some undercutting may occur along this segment (Fig. 5c). At c. 50–60 m depth, the cross-sectional base of the subglacial bedrock is flat and nearly horizontal, resulting in a L-shaped cross-sectional geometry of the bedrock surface underneath the ice. This is also the area where the ice surface starts to bend from a South-West to a West-oriented flow direction together with the development of a niche (*α*) similar to an incipient cirque (Figs 1 and 5). Farther downslope of cross-section (c), the cross-sectional geometry changes again. Above the glacier, the bedrock surface dips at 40°−50° and steepens by a few degrees at the ice surface, thus forming a break-in-slope where the bedrock plunges beneath the glacier (sections (d) and (e) on Fig. 5). Along these sections, the base of the glacier is flat to slightly tilted towards the glacier’s outer side, and the transition to the lateral margin is much more gradual than for the upslope reaches (sections (b) and (c) on Fig. 5).

While the outer glacier is covered by many muon trajectories (Figs 1 and 5), the inner glacier on the northern side of the bedrock ridge is out of the field views of the detectors. Therefore, the elevation of the bedrock underneath the inner glacier has to be constrained through indirect evidence. Here, we use information from the elevation of the bedrock ridge itself (Fig. 5) to infer the elevation of the bedrock underneath the inner glacier. In particular, the bedrock depth along the outer glacier is situated at the same elevation, or even higher, than the ice surface of the straight inner glacier at least along cross-sections ‘d’ and ‘e’ in Fig. 5. This suggests that the underlying bedrock underneath the steep and straight inner glacier is likely to be at a lower elevation than the reconstructed bedrock below the outer glacier, at least along a section perpendicular to the ice flow.

## Discussion

We verify our subglacial bedrock reconstruction within the Eiger headward region (segments I and II) following two pieces of evidence. First, the reconstructed bedrock positions agree with the mapped hillslope walls above the lateral glacier margin (Fig. 5). Second, the reconstructed bedrock positions also match with the domain where the bedrock is partially exposed. This is mainly the case for the bedrock ridge that separates the inner from the outer glacier (cross-sections in Fig. 5). These observations are supporting evidence for a reliable performance of the muon-flux attenuation analysis and the related calibration for our study site (see Methods). The muon radiography method thus yields quantitative information on the ice thicknesses (c. 10–30 m precision) of remote Alpine glaciers, if suitable detector sites such as natural bedrock galleries or tunnels are available underneath the target glacier. We thus propose that the technology of cosmic-ray muon radiography with emulsion detectors offers a quantitative tool that complements established geophysical methods^42^. In the case of the Eiger glacier, the steep bedrock slopes below the glacier even sharpen the density contrasts in the retrieved muon-derived images, while other methods (such as seismic or radio-echo soundings) often fail to yield information with acceptable resolution for similar bedrock geometries. Moreover, our present survey suggests that this novel technology works well in remote and harsh environments with the advantage of the emulsion films as muon detectors. The emulsion films showed an excellent performance in the remote and cold environment during the entire measurement period (spanning several months) without electricity or regular maintenance. This passive and robust feature of the measurement device cannot be attained by electronic detectors such as digital detectors including plastic scintillation and gaseous detectors, which have so far been applied to muon radiography in many prior works^43–47^.

With respect to the Eiger glacier and its erosional mechanisms, the most prominent result of our work is the reconstructed shape of the lateral margin along the outer glacier. In the upper reach of our surveyed area (section (a) on Fig. 5), the shape of the bedrock-ice interface is characterized by a generally smooth transition from the lateral margin to the base of the glacier. In the reach where the glacier begins to curve and where a bedrock niche points to the occurrence and possibly ongoing development of an incipient cirque, the bedrock underneath the lateral glacier margin appears much steeper than the exposed hillslope (sections (d) and (e) on Fig. 5) and almost vertical-to-overhanging in some reaches (sections (b) and (c) in Fig. 5). Such bedrock-slope variations over short distances (few tens to hundred meters) require a strong lateral and headward erosional component to shape the nearly vertically-oriented margin of the subglacial bedrock. Interestingly, this is also the glacier segment where no crevasses are observed on the ice surface. This implies that the glacier is likely under compression along this reach, which provides a precondition for erosion on the lateral margin of a glacier to occur. Along the inner glacier reach, vertical glacial carving has been more efficient than along the outer curved glacier. We based this inference on the observation that the subglacial bedrock under the inner glacier is situated at lower elevations than along the outer curved reach.

The erosional mechanisms of an alpine glacier, related efficiencies and controls thereof have been explored through both empirical observations and numerical investigations over the past decades. This resulted in the overall notion that the local erosional capacity of an Alpine glacier depends on its flow velocity raised to some power, modulated by different controlling factors such as subglacial hydrology, fracture spacing of the underlying bedrock and debris concentration in the basal ice^4,6,7,48–50^. It has also been proposed that the ice-flow velocity and the basal shear stresses are closely related, where shear increases with flow velocity raised to some power^6^. This likewise suggests that subglacial erosion and thus the glacial impact on mountainous landscapes exponentially increase with the basal shear stress at the ice-bedrock interface. Under warm-based glaciers, shear stresses and erosion rates can also be modulated by the subglacial hydrology^7^. In the case of the Eiger glacier, the curved geometry of the glacier reaches along the outer part of segments II and III may additionally result in a highly asymmetric pattern of shear stresses and flow velocities due to the curvature. This is explained by the contrasts in the slopes between the inward and outward segments of a curved glacier. As a consequence, the flow velocity and stress centre line, commonly situated in the middle of a straight glacier, may be shifted toward the inner side of a bend, where the amount of this shift increases with the curvature of the ice flow^51^. We use these mechanisms to explain the lower depth at which the bedrock has been carved along the straight and steep inner reach of the Eiger glacier (Fig. 5).

While the larger bottom shear stresses along the straight inner glacier could be invoked to interpret the lower elevations of the bedrock topography (see cross-sections of Fig. 5), these mechanisms alone are not capable of explaining the nearly vertical lateral dip of the ice-bedrock interface along the southern margin of the outer glacier. Such an erosional component, operating on the side of a glacier, requires substantial shear stresses on the lateral ice margin. This suggests that glacial erosion depends not only on the ice thickness and the slope, but also on the momentum of the flowing ice particularly where glaciers appear under compression (segment II, Fig. 1). Ice flow thus shows similarities with a fluid when considering the erosional mechanisms: both apparently have a vertical and a lateral component where concave banks can experience stronger erosion. This L-shape sculpting acts to widen the glacial thalweg in the lateral direction with a strong over-steepening of the lateral margin. After the glacier retreats, such oversteepened cliffs would be exposed above the glacier and collapse due to the loss of mechanical support or activated hillslope/fluvial processes^52^. This could be a reason why we do not observe this morphology in deglaciated areas. Alternatively, the oversteepened bedrock could represent the headwall of an incipient cirque, which additionally feeds, or starts to feed, the Eiger glacier from the southwest. We base this inference on the plan-view shape of the ice margin, which is nearly straight along segment I (Fig. 1) and then curves towards the headwall in the middle of segment II. Accordingly, the vertical to nearly over steepened glacier margin, which was imaged through the cosmic-ray muon radiography (sections (b) and (c) of Fig. 5) better reflects the erosional work along the cirque headwall through backward erosion.

The search for the controls on cirque wall retreat has received much attention in the past years^12,13,16^, yet with diverging conclusions, which range from bedrock shattering through freeze-thaw cycles^53^, to bedrock carving in response to rotational flow of ice, and to quarrying^16^. In addition, using numerical simulations where glacial erosion has been treated as a function of basal sliding, McGregor *et al*.^16^ proposed that cirques are preferentially formed hundreds of meters below the ELA, and several studies suggested that the position of the cirque floor may reflect the averaged ELA position over multiple glacial-interglacial cycles^16,54,55^. Because our results highlight the occurrence of erosional carving at the inferred incipient cirque, and since the modern ELA (and thus also the long-term averaged ELA) is lower than the elevation of this incipient cirque, we suggest that glaciers are also capable of efficient headwall scouring above the ELA. In addition, the bedrock geometry underneath the cirque does correspond to the shape that is expected if a rotational flow, paired with shear along the lateral margin, is responsible for the occurrence of cirque wall retreat, supporting current views on the erosional mechanisms of glaciers and cirque evolution.

As a summary, we have illustrated a successful application of the cosmic-ray muon radiography, where we reconstructed the bedrock topography under an active glacier over an area of several hundreds of meters. Our results suggest that this technology is capable for mapping the interface between ice and bedrock at a resolution of 10–30 m, suitable for this study. It also allows the imaging of steep and nearly overhanging boundaries between ice and bedrock, which would not be depicted with other geophysical methodologies. From a geomorphological perspective, the results of our survey imply that active glacial backward erosion does occur in the accumulation area above the modern ELA (situated at c. 3000 m a.s.l.^41^) and that this mechanism can be invoked to explain the formation and the long-term evolution of glacial cirques. Further high-resolution constrains on the bedrock topography for high-elevation glaciers will allow us to improve our understanding on the erosional mechanisms at work in cirque environments where glaciers originate.

## Methods

### Emulsion detectors and analysis

Double-side coated emulsion films were used as muon detectors^38^. The thicknesses of the plastic base and emulsion layers were 180 μm and 60 μm, respectively. The emulsion gels, which were produced by the Nagoya University^56^, were poured onto the plastic base at the underground facility at the University of Bern. The produced films were transported to the experiment sites and installed in the detector frames mounted onto the wall of the railway tunnel. In the detector frames, five or six films were layered, and 2-mm-thick stainless steel plates were inserted between each adjacent film^57^. The location and size of the detectors, as well as the exposure time are reported in Table 1.

**Table 1:** Description of detector sites.

	Position (Lng/Lat/Alt)	Facing (azimuth)	Effective area (cm^2^)	Exposure time
ES (Eismeer Station)	8°0′37.72″E 46°34′21.40″N 3159.9 m	239.1°N	1512	9.227 × 10^6^ sec (15 Mar – 30 Jun, 2017)
TA (Tunnel Site A)	8°0′28.37″E 46°34′8.44″N 3186.4 m	260.5°N	1296	1.4245 × 10^7^ sec (30 Jun – 12 Dec, 2017)
TB (Tunnel Site B)	8°0′14.99″E 46°33′56.07″N 3215.8 m	305.1°N	1080	9.229 × 10^6^ sec (15 Mar – 30 Jun, 2017)

The films were chemically developed after extraction and scanned with the automated readout microscopes at the Laboratory for High Energy Physics, at the University of Bern. The microscope consists of a CMOS camera, a motorized microscope stage and illumination^38^. This allows to take tomographic profiles of silver grains in the emulsion layers and to measure the position and direction of incident charged particles^38^. The procedure is as follows: (i) a sequence of silver grains in each emulsion layer is selected as muon track candidates (micro-tracks); (ii) micro-tracks from two layers of a film, which penetrate the plastic base in a straight way, are selected (base-tracks); (iii) base-tracks aligned in consecutive films are identified as muon trajectories, here defined by at least three base-tracks out of the total of five (TB) or six films (ES and TA). The processes (i) and (ii) were performed by means of an in-house developed software^58^, while (iii) was performed with the FEDRA software^59^. Figure 2 shows the incident angle distribution for the reconstructed tracks, where the angular resolution of the tracks is 3–10 milliradian. The number of detected muons was then determined in rectangular bins and converted into a muon flux (cm^−2^ s^−1^ sr^−1^) by normalizing with respect to the scanned area, the exposure time and the solid angle. The inefficiency of the films was estimated to ~10% at base-track level, which leads to the muon detection efficiency of 95–99%. We performed a correction of the measurement results taking the detection efficiency into consideration.

### Flux simulation and calibration

Before reconstructing the bedrock shape, a calibration of the flux attenuation analysis was performed by comparing the observed flux of muons through pure rock with the corresponding simulated flux. TA site provides such data (18 bins from forward direction and 60 bins from backward). The simulation is performed assuming a rock density ρ_rock of 2.68 g cm^−3^, which is the average bulk density of the local limestone, determined from 16 samples (2.68 ± 0.02 g cm^−3^) collected inside the railway tunnel and at the surface^31^. The calculation of the muon flux is based on the energy spectrum model of cosmic-ray muons and the interaction of muons with matter. We employed the spectrum model proposed by Tang^60^ and the muon range for standard rock tabulated by Groom^61^. The topography of the mountain and the glacier surface is taken from a 2 m mesh digital elevation model (Swisstopo© with elevation uncertainty of <3 m) to derive the length of the muon trajectories from the topographic surface to the tunnel sites (L). Since this obstacle length varies within the bin due to the steep topography of the mountain, the bin is further divided into small hundred bins so that the roughness within the subdivided bin can be negligible. For each subdivided bin, the density-length traversed by muons is calculated by multiplying the rock density (ρ_rock) and the length of the muon trajectory (L). The minimum energy of muons (E_min) needed to penetrate this density-length can be looked up from the range table^61^. The muon flux is obtained by integrating the energy spectrum from E_min to infinity. The muon flux values calculated for hundred subdivided bins are then averaged out and set to the representative value of the original rectangular bin.

Figure 3 shows the observed flux as a function of rock thickness and zenith angle. The observed data at the TA site generally agrees with the theoretical curves. The ratio between the observed and simulated flux is found to be 0.94 ± 0.04 (stat), independent of the zenith angle and the rock thickness. A small deviation of this ratio (0.94) from one can be regarded as a bias of this observation due to the systematic uncertainties in the detector efficiency and the muon energy spectrum model. The statistical fluctuation (4%) is comparable to the effect of the intrinsic variability of the bedrock density (1% from rock sample measurements) on the resultant muon flux (3%). In a further analysis, therefore, this difference is calibrated when comparing the observed and simulated flux by multiplying the simulated flux by this factor (0.94).

### Bedrock shape reconstruction

We followed the approach by Nishiyama *et al*.^31^ upon reconstructing the shape of the bedrock underneath the Eiger glacier. The observed muon flux for each bin yields the average bulk density <ρ> of the material confined in the viewing range of the bin. Since the confined region is a mixture of bedrock and ice, the fraction of the bedrock (x) is related to the bulk density of a bedrock (ρ_rock) and that of an ice component ρ_ice = 0.85 g.cm^−3^ by Huss^62^ through the relationship:

$$ < \rho  > =\rho {\rm{\_}}{\rm{r}}{\rm{o}}{\rm{c}}{\rm{k}}\cdot {\rm{x}}+\rho {\rm{\_}}{\rm{i}}{\rm{c}}{\rm{e}}\cdot (1-{\rm{x}})$$

Once x is obtained for each bin, the boundary position can be plotted at a distance Lx from the detector position. Here, a constant density is inferred for the ice because snow and firn exhibit a lower density in only the uppermost 10 m of the glacier^62^, with significant density changes only occurring in the varying snow cover layer or in the presence of crevasses^63^, which are almost absent along the reconstructed glacier reach (Fig. 1). The resulting uncertainty is in the order of the DEM error^63^ and thus negligible compared to the scale of our observation. The three-dimensional representation and error estimation are performed as follows. First, the observed muon flux dataset is multiplied to hundred synthetic datasets by adding a statistical fluctuation for each bin. Specifically, a random variable following a Gaussian distribution with a standard deviation of $\sqrt{N}$ is added when the number of muons in the bin is N. Subsequently, the ice-rock boundary position is calculated for each flux data in every synthetic dataset and plotted in a 3D space. In the end the plotted point clouds are re-sampled in a cylindrical coordinate system with the axis parallel to the glacial flow direction (south-west) with a division of Δz = 40 m along the axis and Δϕ = 12° in azimuth. For each divided cylindrical bin (z, ϕ), the best position of the ice-bedrock boundary is given by taking the average of the radial coordinates of the points. The error of the boundary position is given by their standard deviation (yellow bands in Fig. 5). A synthetic data reproduction was introduced so that the magnitude of the statistical fluctuations is properly taken into account after re-sampling. Cylindrical bins, which contain less than 10 points, were neglected as lack of statistics and resolving power.
